# Editorial: Deciphering the root nodule microbiome: implications for legume fitness and stress resilience

**DOI:** 10.3389/fmicb.2025.1634838

**Published:** 2025-06-09

**Authors:** Esther Menéndez, Clarisse Brígido

**Affiliations:** ^1^Departamento de Microbiología y Genética, Universidad de Salamanca, Salamanca, Spain; ^2^Institute for Agribiotechnology Research (CIALE), Villamayor, Salamanca, Spain; ^3^Unidad Asociada Grupo de Interacción Planta-Microorganismo, Universidad de Salamanca-IRNASA-CSIC, Salamanca, Spain; ^4^MED—Mediterranean Institute for Agriculture, Environment and Development & CHANGE—Global Change and Sustainability Institute, Institute for Advanced Studies and Research, Universidade de Évora, Évora, Portugal

**Keywords:** rhizobia, endophytes, microbiome, legumes, plant growth promotion, symbiosis, plant protection, biological nitrogen fixation (BNF)

The legume-rhizobia symbiosis is a key plant-microbe interaction crucial for biological nitrogen fixation. Historically, it was believed that only nitrogen-fixing rhizobia colonized legume root nodules, influencing decades of research. This mutual relationship, in which rhizobia provide usable nitrogen to plants in exchange for carbon, plays a vital role in soil fertility and sustainable agriculture (Goyal et al., 2021). However, recent advances in molecular microbiology and the application of high-throughput omics technologies have challenged this classical view. Studies employing next-generation sequencing and metagenomic approaches have revealed that legume root nodules are more than just rhizobia habitats (Hakim et al., 2020; Liu et al., 2022; Zhang et al., 2018). Instead, they are complex microecosystems inhabited by diverse microbial consortia (Martínez-Hidalgo and Hirsch, 2017). In addition to rhizobia, these nodules also contain non-rhizobial bacteria, which were once dismissed as contaminants but are now recognized as consistent and potentially functional members of the nodule microbiome. These non-rhizobia include a wide range of bacterial taxa, some of which possess plant growth-promoting traits such as phosphate solubilization, phytohormone production, or pathogen suppression (Hassen et al., 2025). Their presence suggests that they may play synergistic or complementary roles in enhancing nodule function, influencing nutrient exchange, and contributing to plant resilience under stress (Etesami, 2022). Yet, despite these discoveries, the ecological roles, mechanisms of colonization, and interactions between these non-rhizobial microbes with rhizobia and host plants remain poorly understood.

This Research Topic brings together recent research exploring the microbial diversity, ecological functions, and agricultural applications of root nodule microbiomes, significantly expanding the traditional understanding of legume-rhizobia symbiosis. The Research Topic includes original research on rhizobia and non-rhizobia, with an emphasis on molecular and amplicon-based metagenomics approaches, and a review article which consolidates findings on the prevalence and functionality of non-rhizobia, emphasizing their role in enhancing plant performance when co-inoculated with rhizobia.

The study by Gao et al. investigated how rhizobial populations associated with *Astragalus mongholicus* vary across different geographic and environmental conditions. The rhizobial communities in NE China are genetically diverse and composed of multiple genera, with limited gene flow between regions suggesting independent evolutionary paths. The plant's ability to form symbiosis with various rhizobial species may help it adapt and efficiently fix nitrogen in different environments. Adan et al. assessed the microbial diversity within the root nodules of three *Desmodium* species (*D. uncinatum, D. intortum*, and *D. incanum*) used in push-pull cropping systems in Kenya. The authors employed an amplicon-based metagenomic approach to target bacterial and fungal communities, revealing the predominance of *Bradyrhizobium* and *Fusarium*. Although *Desmodium* species host similar root nodule microbiomes, *D. uncinatum* showed a higher presence of genes related to energy and amino acid biosynthesis, suggesting it may contribute more effectively to soil health and agroecosystem functioning.

Yuan et al. investigated how nitrogen content influences the diversity and community structure of nitrogen-fixing microorganisms in the root nodules and rhizosphere soils of three *Alnus* species with different chromosome ploidies. Using high-throughput sequencing of the 16S rRNA and *nifH* genes, the authors compared the microbial communities in *A. glutinosa, A. formosana*, and *A. cremastogyne*. In parallel, nitrogen levels in the nodules and the rhizosphere of these plants were also measured. They found that root nodules contained higher total and nitrate nitrogen, but lower ammonium nitrogen compared to rhizosphere soils. *A. glutinosa* exhibited the highest microbial diversity and nitrogen-fixing potential, with significantly greater abundance of *Frankia* and associated nitrogen cycle functions than the other two species. The study concludes that nitrogen content, particularly total nitrogen, strongly influences the abundance and diversity of nitrogen-fixing microbes like *Frankia*, highlighting *A. glutinosa* as a promising species for enhancing soil fertility through biological nitrogen fixation.

Ali et al. investigated the potential of nodule-inhabiting *Paenibacillus* species, particularly those within the *Paenibacillus polymyxa* complex, as biocontrol agents and plant growth promoters. The authors screened strains isolated from legume root nodules for their ability to inhibit five major phytopathogenic fungi and evaluated their production of antifungal compounds like fusaricidins. Additionally, plant growth-promoting traits traits such as indole acetic acid production, phosphate solubilization, nitrogen fixation, and plant colonization were examined. It was found that several of these strains possess broad-spectrum antifungal activity, can effectively colonize wheat plants, promote plant growth, and control fungal diseases *in planta*, making them promising candidates for sustainable agriculture as biocontrol agents and biofertilizers.

The systematic review by Hnini and Aurag consolidates findings on the prevalence and functionality of non-rhizobia, emphasizing their role in enhancing plant performance when co-inoculated with rhizobia. While traditionally thought to be solely inhabited by rhizobia, legume nodules also host a consistent and beneficial community of other bacteria, such as *Bacillus, Pseudomonas* and *Paenibacillus*. Although non-rhizobial endophytes (NREs) do not induce nodule formation, they enhance plant growth and resilience through mechanisms like nitrogen fixation, phosphate solubilization, siderophore production, and stress tolerance. The review also underscores that co-inoculation of legumes with both rhizobia and NREs generally leads to improved plant growth and yield compared to inoculation with rhizobia alone. It emphasized the specificity of plant-NRE associations, particularly in *Phaseolus*, and notes the consistent predominance of *Bacillus* and *Pseudomonas* across diverse legume species. The findings have important implications for sustainable agriculture, pointing out that targeted microbial inoculation strategies can enhance nutrient uptake, tolerance to different stresses, and overall crop performance and productivity under diverse environmental conditions.

In conclusion, these studies collectively demonstrate that the root nodule environment harbors a high complexity of functionally active microbial communities with great potential for achieving advances toward sustainable agriculture. However, a critical knowledge gap remains in understanding the specific roles and interactions of non-rhizobial microbes. Future research should prioritize functional analyses to uncover how these microbial communities influence legume-rhizobia symbiosis, plant health, and agrosystems productivity.
